# Arthroscopic Debridement of the Posterior Compartment of the Knee after Total Knee Arthroplasty

**DOI:** 10.1155/2014/568417

**Published:** 2014-08-18

**Authors:** Tsuyoshi Ohishi, Tomotada Fujita, Daisuke Suzuki, Kazufumi Yamamoto, Hiroki Ushirozako, Yukihiro Matsuyama

**Affiliations:** ^1^Department of Orthopaedic Surgery, Enshu Hospital, 1-1-1 Chuo, Naka-ku, Hamamatsu, Shizuoka 430-0929, Japan; ^2^Department of Orthopaedic Surgery, Hamamatsu University School of Medicine, Hamamatsu, Shizuoka 431-3192, Japan

## Abstract

Arthroscopic debridement of the posterior compartment of the knee after total knee arthroplasty is difficult because it is tough to obtain intercondylar notch views. Herein, we performed arthroscopic debridement of the posterior compartment of an infected knee after total knee arthroplasty by using a transseptal portal in a 62-year-old woman with rheumatoid arthritis. Palpation of anatomical landmarks and posterior capsule protection are important for safe creation of a transseptal portal following to making 2 posterior portals.

## 1. Introduction

Pyogenic arthritis is one of the devastating complications of total knee arthroplasty (TKA). Arthroscopic debridement and irrigation is one of the treatment options available for pyogenic arthritis after TKA in selected patients who do not present with radiographic evidence of loosening of components [1–4]. However, arthroscopic debridement and synovectomy of the posterior compartment of the knee with prosthesis are difficult. In this report, we performed debridement and synovectomy of the posterior compartment of an infected knee following TKA using a posterior transseptal portal in a 62-year-old woman with rheumatoid arthritis. To our knowledge, this is the first report on successful arthroscopic procedures performed in the posterior compartment of the prosthetic knee using posterior transseptal portal.

## 2. Case Report 

**Figure 1:**
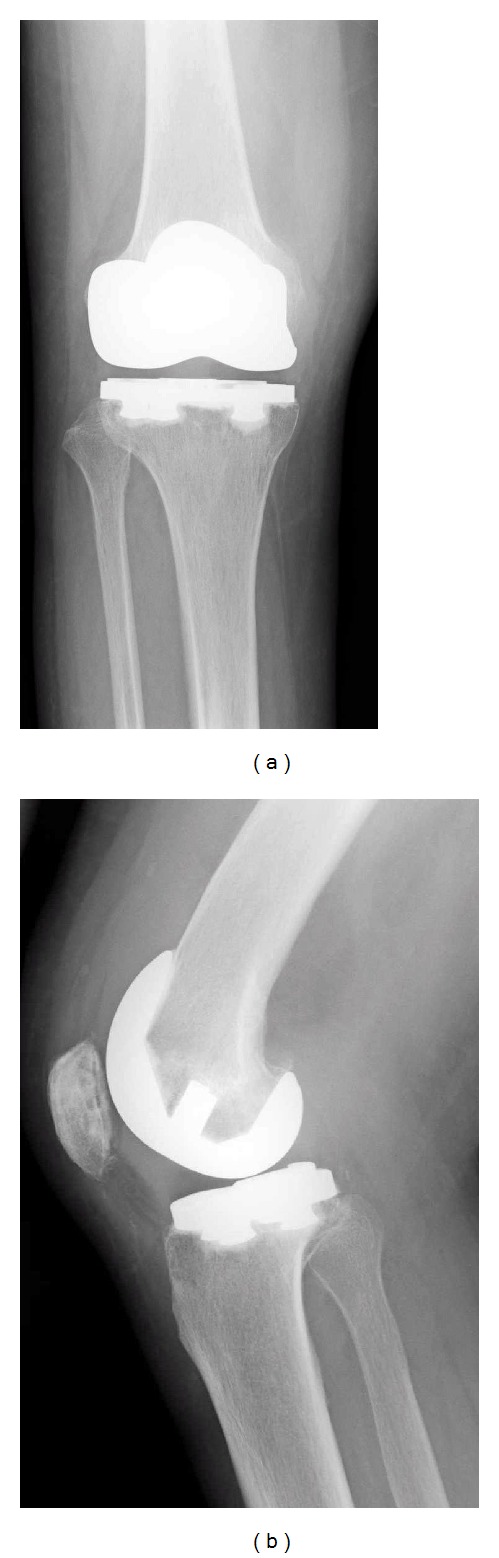
Anteroposterior (a) and lateral (b) radiographs of the patient's right knee at the first visit. No signs of infectious loosening or osteolysis around the prosthesis were detected.

A 62-year-old woman, diagnosed with rheumatoid arthritis at 28 years of age, was referred to our hospital with a history of right knee pain for 5 days. She had undergone posterior cruciate-retaining TKA (Zimmer, Warsaw, IN, USA) of the right knee 10 years previously at another hospital. Methotrexate (6 mg/week), prednisolone (2.5 mg/day), and bucillamine (200 mg/day) had been administered for 4 years. On examination, her right knee was tender, swollen, and red, with limited range of motion. Laboratory findings revealed a leukocyte count of 10200 cells/*μ*L and C-reactive protein level of 8.37 mg/dL. Gram-positive cocci were identified from the aspirated joint fluid of the right knee. The patient was diagnosed with pyogenic arthritis post-TKA. Because of the acute onset and absence of radiographic loosening or osteolysis around the prosthesis (Figures 1(a) and 1(b)), we tried to retain the components by arthroscopic debridement without removal of them.

**Figure 2:**
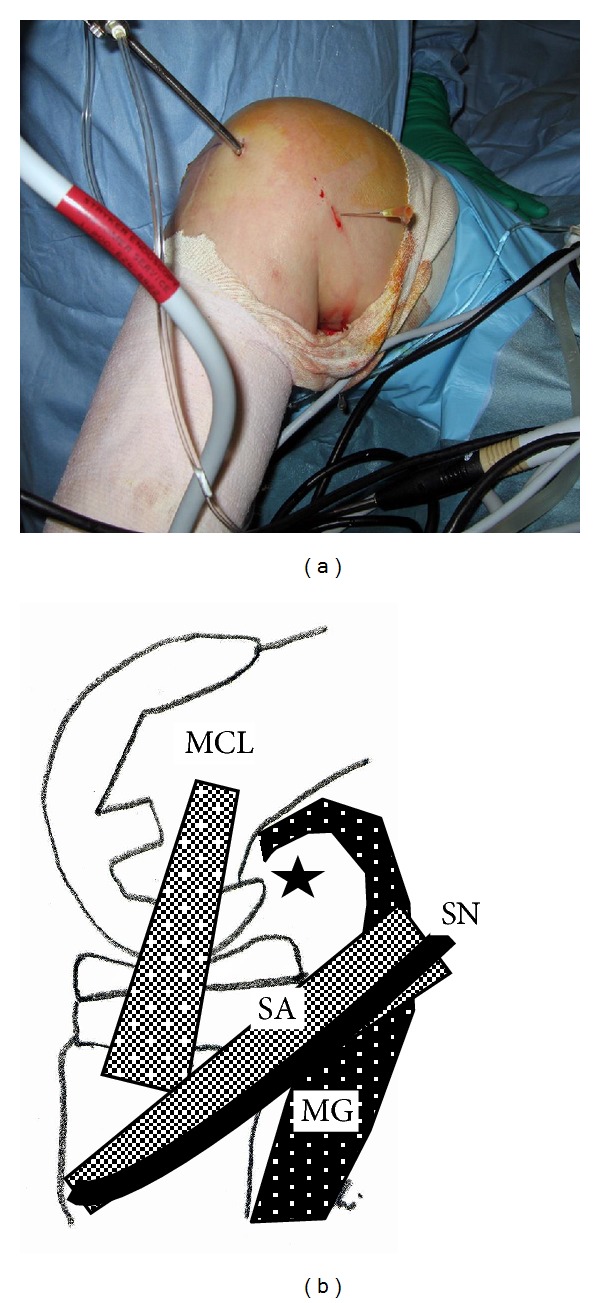
(a) Operative procedure to determine the site of the posteromedial portal. A 22-gauge needle was inserted at the edge just posterior to the medial aspect of the femoral component. Note the drops of irrigation solution dripping from the inserted needle. (b) The black star indicates the position of the posteromedial portal. MCL: medial collateral ligament; MG: medial head of the gastrocnemius muscle; SA: sartorius; SN: saphenous nerve.

**Figure 3:**
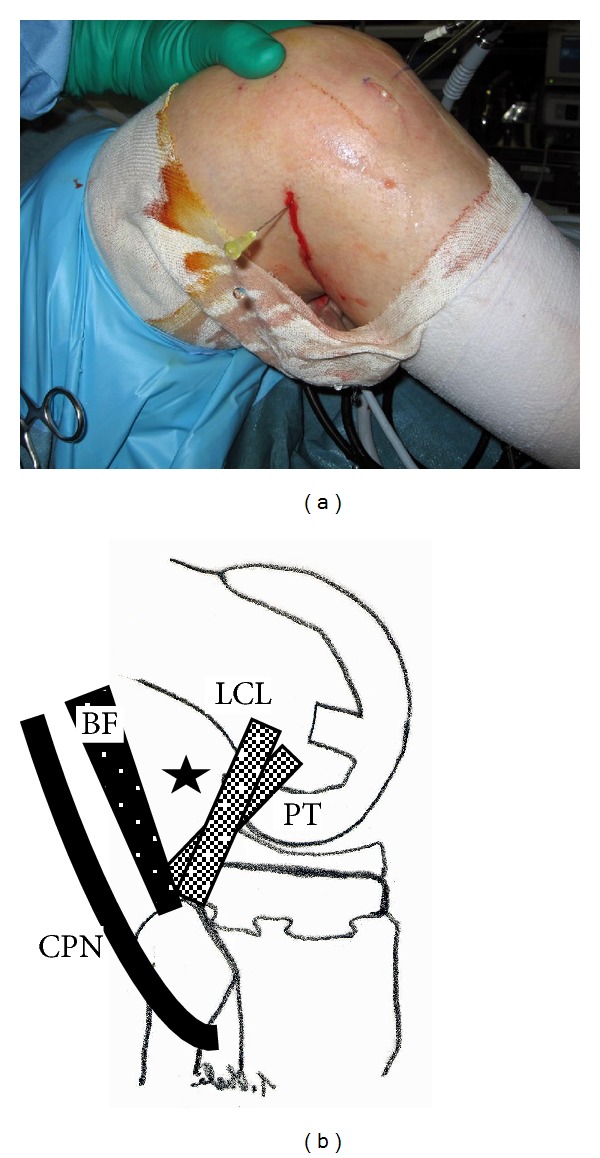
(a) Operative procedure to determine the site of the posterolateral portal. A 22-gauge needle was inserted at the edge just posterior to the lateral aspect of the femoral component. (b) The black star indicates the position of the posterolateral portal. BF: biceps femoris; CPN: common peroneal nerve; LCL: lateral collateral ligament; PT: popliteus tendon.

**Figure 4:**
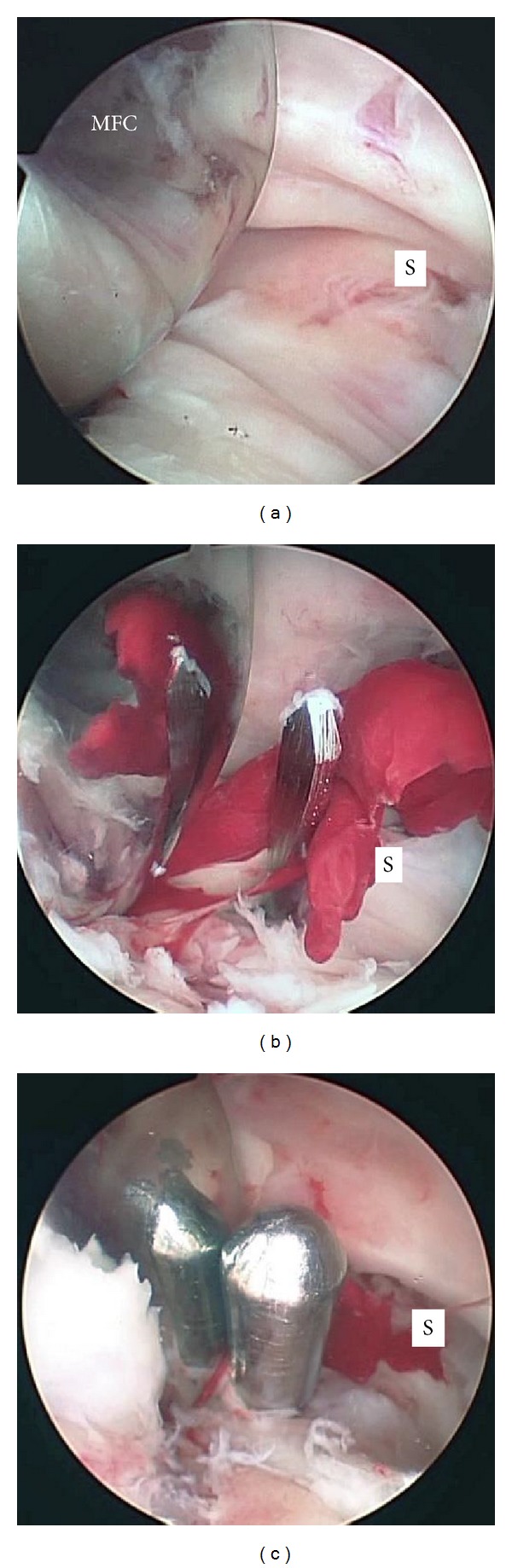
Arthroscopic views of the posterior compartment. The posterior septum was confirmed when viewed from the posteromedial portal (a). The septum was perforated by using a 3.0 mm Kirschner wire that was inserted from the posterolateral portal protected by a sheath (b). A 4.0 mm rod from the posterolateral portal was inserted into the initial hole created in the septum by using the Kirschner wires (c). MFC, medial condyle of the femoral component; S, septum.

**Figure 5:**
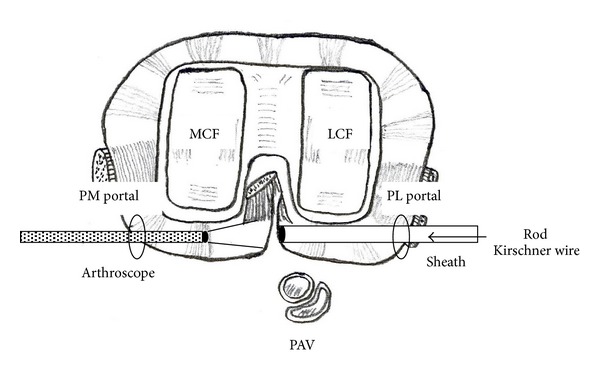
This schema demonstrates the method to create a posterior transseptal portal. A sheath inserted from the posterolateral portal is pushed to the septum to protect popliteal vessels that lie immediately behind the lateral posterior capsule. Viewing the septum through the posteromedial portal, it is perforated first by using a 1.5 mm Kirschner wire followed by a 3.0 mm Kirschner wire through the sheath from the posterolateral portal (arrow). PM portal, posteromedial portal; PL portal, posterolateral portal; PAV, popliteal artery and vein; MCF, medial condyle of the femoral component; LCF, lateral condyle of the femoral component.

**Figure 6:**
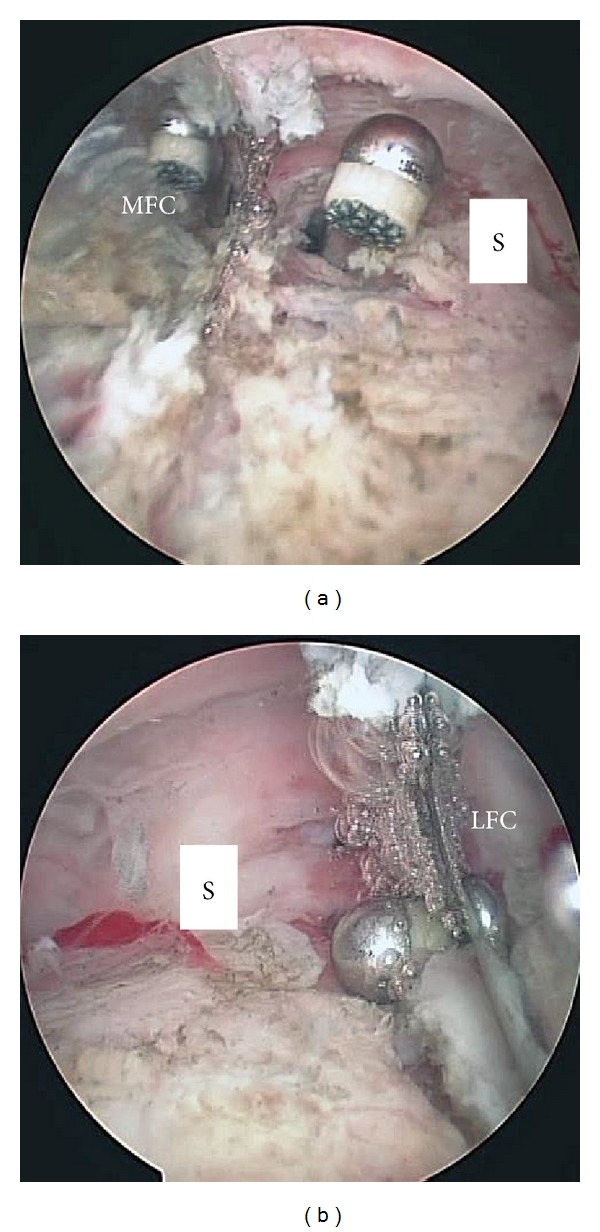
Arthroscopic views during debridement and synovectomy in the posterior compartment. Debridement and synovectomy in the posteromedial (a) and posterolateral (b) compartments were performed with a radiofrequency abrader that was introduced from the posterolateral (a) and posteromedial (b) portal through the posterior transseptal portal. S, septum; MCF, medial condyle of the femoral component; LCF, lateral condyle of the femoral component.

With the patient under general anesthesia, the affected knee was flexed to >90° on the operating table with the use of a foot stopper. Moderate synovitis and massive debris were observed in the suprapatellar pouch and medial and lateral gutters. Arthroscopic debridement and synovectomy of the anterior compartment of the knee were performed through the 2 routine anterior portals. In routine cases, in order to establish the posteromedial and posterolateral portals, the arthroscope is passed through the intercondylar notch [5]. However, in TKA patients, the polyethylene insert interfered with the advance of the arthroscope to the posteromedial or posterolateral compartments through the intercondylar notch. Therefore, we made 2 posterior portals guided by palpable anatomical landmarks on the posteromedial and posterolateral aspects of the knee. To make the posteromedial portal, a 22-gauge needle was inserted immediately behind the posterior edge of the medial aspect of the femoral component at approximately 0.5 cm above the femorotibial joint line to avoid saphenous nerve injury. Drops of irrigation solution from the needle confirmed its position (Figures 2(a) and 2(b)). A 1 cm skin and capsule incision, simultaneously and without direct visualization, was made immediately anterior to the 22-gauge needle by using a number 11 blade. A posterolateral portal was then created in the same manner as the posteromedial portal by using a 22-gauge needle to determine the posterolateral portal site. Palpation of the common peroneal nerve and fibular head could be helpful in avoiding common peroneal nerve injury. Similarly, a 22-gauge needle was inserted in the lateral aspect of the femoral component first, and then the needle insertion site was moved posteriorly to find the posterior edge of the lateral aspect of the femoral component (Figures 3(a) and 3(b)). Once the posterolateral portal was created, a 4.0 mm rod with a sheath (rod/sheath) was inserted through the posterolateral portal to the septum. The arthroscope was then introduced from the posteromedial portal to view the medial aspect of the posterior septum (Figure 4(a)). We could determine the puncture site in the septum by pushing the septum from the opposite site with the rod/sheath. While keeping the medial side of the septum in view with the arthroscope introduced through the posteromedial portal, a 1.5 mm Kirschner wire, followed by a 3.0 mm Kirschner wire, was pushed to the septum through the sheath from the posterolateral portal, and then the septum was perforated (Figure 4(b)). A rod was then pushed through the sheath from the posterolateral portal to the posteromedial portal via a transseptal portal (Figures 4(c) and 5). Once a transseptal portal was created, the arthroscope and instruments were easily interchanged through the 2 posterior portals according to the posterior “back and forth” approach presented by Louisia et al. [6], and debridement and synovectomy of the posterior compartment were completed (Figures 6(a) and 6(b)).

Inlet and outlet tubes were inserted into the knee for continuous closed irrigation drainage after the arthroscopic procedure. Postoperatively, a daily dose of 3 g of cefazolin sodium was administered intravenously for 2 weeks. Methicillin-sensitive* Staphylococcus aureus* was identified on the culture plate. Continuous closed irrigation drainage was performed for 9 days. The patient had an uneventful postoperative recovery. She was free of knee pain with no evidence of recurrence 20 months postoperatively.

## 3. Discussion

In the treatment of pyogenic arthritis after TKA, it is desirable that components be removed if loosening of the components is evident on the radiographs. However, debridement or synovectomy through an open or arthroscopic procedure is recommended in selected patients if neither signs of ostitis nor evidence of a loose prosthetic component is confirmed on radiographic evaluation [1–4, 7–9]. Success rates of arthroscopic irrigation and debridement for infected TKA have been lower than those of the open procedure [4]. This is partly because debridement or synovectomy of the posterior compartment cannot be accomplished by performing routine arthroscopic procedures [3, 4]. When synovectomy or debridement of the posterior compartment is needed in a TKA patient, removal of the polyethylene insert and replacement of the insert is usually employed to eradicate infected synovia in the posterior compartment [8]. We introduced the procedure for debridement and synovectomy in the posterior compartment of the knee after TKA to minimize invasiveness. Posteromedial and posterolateral views through the intercondylar notch should be routinely obtained when creating the 2 posterior portals. However, the polyethylene insert, especially with the tibial post in the case of posterior cruciate-sacrificing TKA, interferes with the advance of the arthroscope to the posterior compartment [3]. If the arthroscope can be advanced to the posteromedial or posterolateral compartment through the space between the metal femoral component and polyethylene insert in cases with posterior cruciate-retaining TKA, damage from the arthroscope, polyethylene insert, or femoral component is possible when creating the 2 posterior portals. If arthroscopic debridement or synovectomy of the posterior compartment of the infected knee after TKA can be achieved, indications for an arthroscopic procedure for an infected TKA may be wider than ever since there are more advantages with arthroscopic debridement than with the open procedure, such as an early recovery of knee function after the surgery. Regarding the technique for creating 2 posterior portals, the methods described in previous reports are not applicable in patients with TKA in situ since it is not possible to obtain an intercondylar notch view [5, 6, 10, 11]. Arthroscopic debridement of prosthetic knees by using 2 posterior portals in addition to anterior portals also has been reported previously [12, 13]. However, details of the exact sites of the posteromedial and posterolateral portals were not indicated. Additionally, the authors did not mention whether a transseptal portal was created to debride the posterior compartment completely in cruciate-retaining TKA in which the posterior septum persisted. The most important step of our procedure is the palpation of landmarks, including the posterior edges of the medial and lateral aspects of the femoral component, fibular head, and common peroneal nerve. According to Ahn et al., the distance from the saphenous nerve to the posteromedial portal was 26.1 mm with the knee in 90° flexion [14]. The saphenous nerve moved posteriorly with flexion of the knee and moved even further with the expansion of the posteromedial capsule during arthroscopic surgery, since the posteromedial compartment was larger than the posterolateral compartment in the expanded knee with the irrigation solution [15]. The distance between the posterolateral portal and common peroneal nerve at 90° knee flexion was relatively wide and reported as being 40 mm by Pace and Wahl [16] and 25.4 mm by Ahn et al. [14]. Ahn et al. also recommended palpating the fibular head as a landmark to create a posterolateral portal if the posterolateral capsule was not visible in the intercondylar posterior view [14]. However, operative procedures during primary TKA altered the relationship between the 2 posterior portal sites and important nerves because of the possibility of adhesion or contracture around the knee. To avoid saphenous nerve and common peroneal nerve injuries, a 22-gauge needle should be inserted at the femoral component first and the insertion site gradually moved posteriorly; the posterior edge of the medial and lateral aspects of the femoral component should then be found. More attention should be paid to posterior capsule injury in the knees of patients who are undergoing TKA when creating a transseptal portal. One of the advantages of our procedure for creating a transseptal portal is that it can be done when the septum is monitored from the posteromedial portal for confirming the proper site, and the sheath can protect the posterolateral capsule where the popliteal vessels are located immediately behind it [17]. To avoid popliteal vessel injury, the posterior transseptal portal should be created via the inferoanterior aspect of the septum. This is the first report that describes the method during an arthroscopic procedure to access the posterior compartment of the knee after TKA using posterior transseptal portal.
